# The *Journal of Orthodontics*: A cross-sectional survey of British Orthodontic Society members

**DOI:** 10.1177/1465312520988549

**Published:** 2021-02-05

**Authors:** Rosalind Jennings, Jadbinder Seehra, Martyn T Cobourne

**Affiliations:** Department of Orthodontics, Centre for Craniofacial Development & Regeneration, Faculty of Dentistry, Oral & Craniofacial Sciences, King’s College London, London, UK

**Keywords:** survey, journal, British Orthodontic Society

## Abstract

**Objective:**

To survey the opinion of British Orthodontic Society members on the *Journal of Orthodontics*.

**Design:**

Data collection involved an anonymous cross-sectional online SurveyMonkey™ questionnaire.

**Methods:**

An email invitation to complete the survey was sent to the 1842 members of the British Orthodontic Society on 9 June 2020 with a follow-up reminder on 15 July 2020. The invitation contained a brief description and online link to the questionnaire, which was active between 9 June and 9 August 2020. The 15-item questionnaire covered frequency of reading, preferred format, likes and dislikes, and what changes might improve the *Journal*. Data were analysed for the membership as a whole using simple descriptive statistics.

**Results:**

In total, 310 individuals completed the questionnaire, representing a response rate of 17% with 74.2% (n = 230) reporting reading at least one article per issue. The most popular way of reading the *Journal* (77.4%, n = 240) was through the distributed print copy. Overall, 63.6% (n=197) rated the *Journal* as excellent and 35.2% (n = 109) as satisfactory, with only 1.3% (n = 4) responding that it was poor. The scientific and clinical articles were the most popular aspect of the *Journal* and 90.3% (n = 280) of respondents felt the *Journal* content was relevant to their current clinical practice. Respondents were also given the opportunity to make additional free-text comments; and themes that emerged included a wish for more clinical content, more online interaction with authors through webinars and continued professional development.

**Conclusion:**

The *Journal of Orthodontics* is perceived as being relevant to current clinical practice by members of the British Orthodontic Society and has high-level satisfaction. There is a desire for more online interaction with the membership as part of its role within the society. However, the overall response rate was low and therefore a high risk of potential bias associated with this survey.

## Introduction

The *Journal of Orthodontics* is an international peer-reviewed academic journal with a global circulation, publishing high-quality, clinically orientated and relevant original research that underpins evidence-based orthodontic care (SAGE, 2020). The *Journal* is published quarterly and is composed of numerous features, including occasional editorials, original research articles, clinical articles and case reports, statistical features, summaries of recent developments in the relevant orthodontic and non-orthodontic literature, book reviews and correspondence to the editor. The *Journal* is currently published by SAGE Journals and edited by Martyn Cobourne who is assisted by a team of associate editors, sub-editors, statistical advisors and an international editorial board.

The *Journal of Orthodontics* is the official journal of the British Orthodontic Society and its origins are embedded in the history of the Society (British Orthodontic Society [BOS], 2020a). It began as *Transactions of the British Society for the Study of Orthodontics*, becoming the *British Journal of Orthodontics* in 1974 and the *Journal of Orthodontics* in 2000 (Jones, 1999). As the sole national representative of orthodontists in the United Kingdom and a registered charity, the British Orthodontic Society promotes the study and practice of orthodontics through research and education, with the aim of maintaining and improving professional standards (BOS, 2020b). The *Journal of Orthodontics* plays a key role in this process. There are currently 1842 members of the British Orthodontic Society, with general membership categories including specialist orthodontists, non-specialists, trainees, technicians, traders and overseas members. Among practitioners, individuals can also choose to join one or more groups, which include the Orthodontic Specialist Group (OSG; n = 727 members) Consultant Orthodontist Group (COG; n = 348 members) Training Grades Group (TGG; n = 292 members), Practitioner Group (PG; n = 277 members), University Teachers Group (UTG; n = 65 members) and Community Group (COM; n = 16 members). All members of the British Orthodontic Society receive a hard copy of the *Journal*, which can also be accessed online from the British Orthodontic Society website by signing in as a member. There is also continued professional development offered in relation to articles within each issue and mediated through the society website. A key responsibility for the editorial board is to produce the four print editions per year, while maintaining academic quality and standards and ensuring that ongoing content and features are relevant and continue to appeal to the readership. For this reason, it is important to engage with the readership and canvass opinion and feedback on how the *Journal* is perceived and how it might change moving into the future.

The aim of the present study was to survey members of the British Orthodontic Society in relation to the content and organisation of the *Journal of Orthodontics* in order to help establish current subscribers’ perceptions of and opinions of the Journal in its current form.

## Methods

Data collection involved the use of an anonymous, descriptive, cross-sectional, online, questionnaire-based survey (Appendix 1). Approval was requested from the British Orthodontic Society Clinical Governance Committee to allow distribution of the survey among its members and this was granted on 4 May 2020. The 15-item questionnaire was developed through consultation with the editorial board of the *Journal of Orthodontics* and was based upon a number of perceived key features of the *Journal* for the readership. An email invitation to complete the survey was sent to all 1842 members of the British Orthodontic Society on 9 June 2020 with a follow-up reminder email invitation on 15 July 2020. The criteria to receive an invitation were: (1) member of the British Orthodontic Society and therefore in receipt of the *Journal of Orthodontics* quarterly; and (2) in possession of a valid email address. The email invitation contained a brief description of the survey’s purpose and an online link to the questionnaire, which was active between 9 June 2020 and 9 August 2020. The questionnaire was created using the online survey provider SurveyMonkey™. The secretary of the British Orthodontic Society distributed the invitation and reminder emails including the survey link to the membership, with all questionnaire responses processed anonymously within SurveyMonkey™. Hence, the confidentiality of all responders was maintained throughout all stages of the study.

Data were obtained from the SurveyMonkey^™^ website, entered into Microsoft Excel and analysed for the membership group as a whole. Descriptive statistics were obtained for all 15 items within the questionnaire.

## Results

A total of 310 individuals completed the questionnaire out of a combined British Orthodontic Society membership of 1842, representing a response rate of 17%. All respondents completed the questionnaire in its entirety and spent a typical time of 4 min to complete it. The highest number of respondents (43%, n = 134) identified themselves as specialist practitioners, with 33% (n = 102) identifying as consultant orthodontists and 11% (n = 34) as members of the training grades. In addition, 13% (n = 40) classified themselves as ‘other’ (Table 1).

**Table 1.** Characterisation as ‘other’.

Dentist with a special interest in orthodontics	24
Retired	6
Associate specialist	4
Community orthodontist	3
Academic	2
Dental Core Trainee (DCT)	1

In terms of general interest in *Journal* content, the majority of responders (74.2%, n = 230) stated that they usually read at least one article in each edition that they received, while 18.7% (n = 58) and 0.6% (n = 2) only read articles in a couple or only a single edition per year, respectively. Among the remainder, 4.2% (n = 13) only look at the table of contents and 2.3% never even open it! In terms of the number of editions that are produced per year, the majority of responders (57.1%, n = 177) would like to see an increase, while 42.9% (n = 133) were happy with the current production of one issue every three months.

There are now multiple methods available to read academic journals but the vast majority of respondents (77.4%, n = 240) still read a hard copy; however, 5.8% (n = 18) now read the digital copy, while 15.5% (n = 48) read the *Journal* using both formats. A small number of respondents (1.3%, n = 4) stated that they used neither method to read the *Journal*. When asked if they would prefer to receive a digital copy of the *Journal* each quarter, the majority of respondents (62.6%, n = 194) said no, they would not.

**Figure 1.**
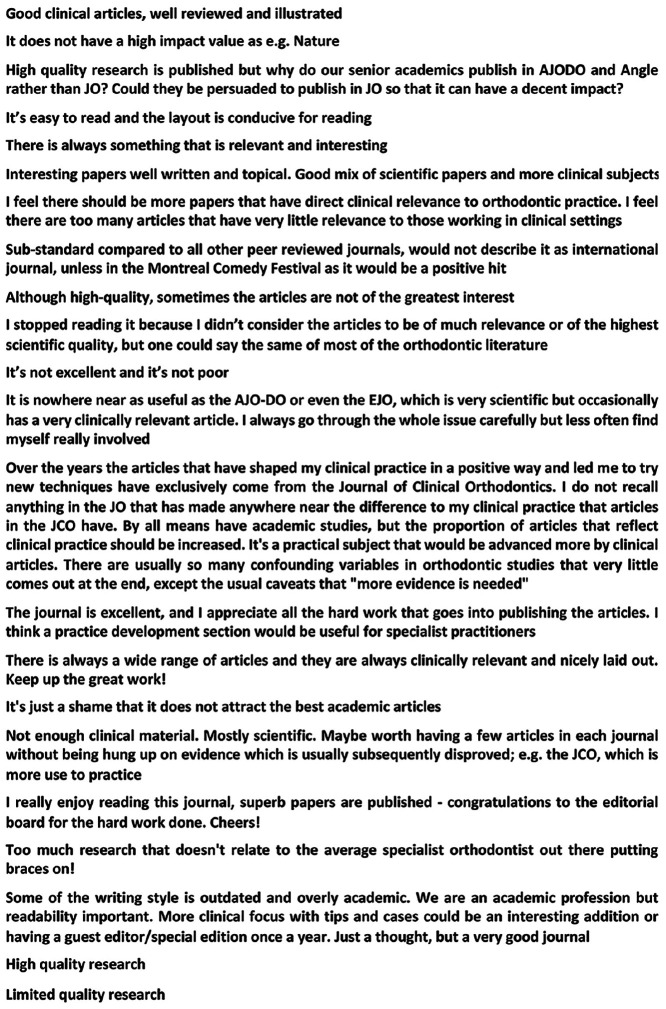
How do you rate the *Journal of Orthodontics*? A sample of representative comments (from a total sample of n = 173).

**Figure 2.**
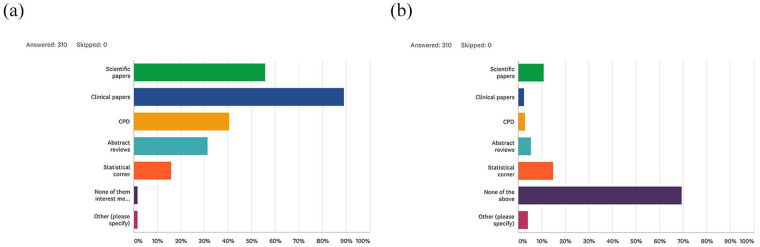
(a) What do you like best about the *Journal of Orthodontics*? (b) What do you dislike about the *Journal of Orthodontics*?

**Figure 3.**
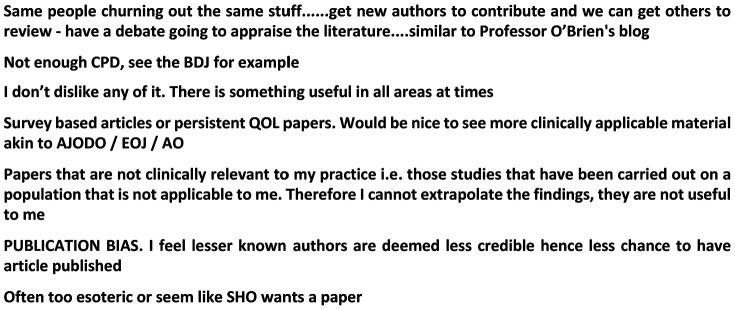
What do you dislike about the *Journal of Orthodontics*? A sample of representative comments (from a total sample of n = 13).

The *Journal* was generally rated positively by respondents, with 63.6% (n = 197) stating that it was excellent, 35.2% (n = 109) satisfactory and only 1.2% (n = 4) suggesting that it was poor. There were also (173 individual text responses to this question and a selection of these are shown in Figure 1. The survey also asked which parts of the *Journal* people liked best and which were disliked (Figure 2a and b). Interestingly, the clinical and scientific articles proved to be the most popular in terms of responses, with only a very small percentage of respondents stating there was rarely anything of interest. In terms of things that were disliked, most respondents (69.4%, n = 215) did not identify any specific sections in the current *Journal* layout. A total of 4.2% (n = 13) did suggest that there were other things they did not like, and a selection of these responses is shown in Figure 3.

The *Journal* has produced a number of supplements in addition to the normal four issues over the last few years, with subject areas including medical problems and orthodontics, lingual appliances, temporary anchorage devices and contemporary issues in orthodontics. When the readership was asked if it would like to see more supplements, there was a fairly even split between those that would (51.3%, n = 159) and those that would not (48.7%, n = 151). When asked if they had a favourite supplement from the last few years, most respondents did not (83.2%, n = 258). When the 60 comments made by responders in relation to this question were analysed, the medical problems in orthodontics and lingual appliances supplements were the most popular supplements.

A further important aspect of the *Journal* is the perceived relevance that it has in terms of clinical practice. The vast majority of respondents (90.3%, n = 280) felt that it was relevant to their clinical practice.

The *Journal* also carries a limited number of advertisements, which are administered by the British Orthodontic Society, and just over half of respondents (63.6%, n = 197) felt that these were useful. However, 26.1% (n = 81) stated that they did not like to see advertisements and 10.3% (n = 32) felt that the *Journal* should not carry advertisements.

**Figure 4.**
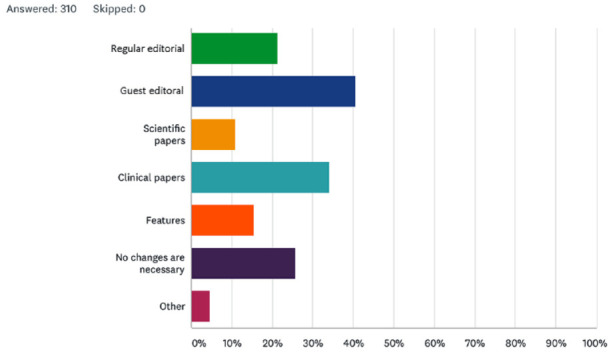
Is there anything specific you would like to see changed in the *Journal of Orthodontics*?

**Figure 5.**
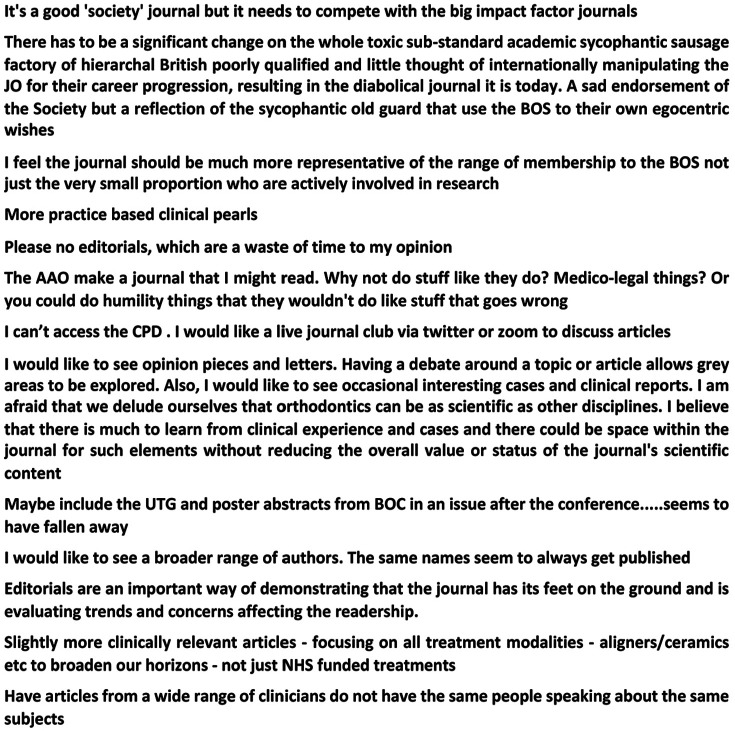
Is there anything you would like to see changed in the *Journal of Orthodontics*? A sample of representative comments (from a total sample of n = 48).

The survey went on to ask if there was anything specific that respondents would like to see changed in the *Journal of Orthodontics* (Figure 4). It can be seen that 21.3% (n = 66) would like to see a regular editorial and 40.7% (n = 126) would like the editorial to feature guest writers. Interestingly, one-quarter of respondents (25.8%, n = 80) did not feel anything should be changed. Respondents were given the opportunity to make specific comments in this section and 48 did so. A selection of these comments is included in Figure 5.

**Figure 6.**
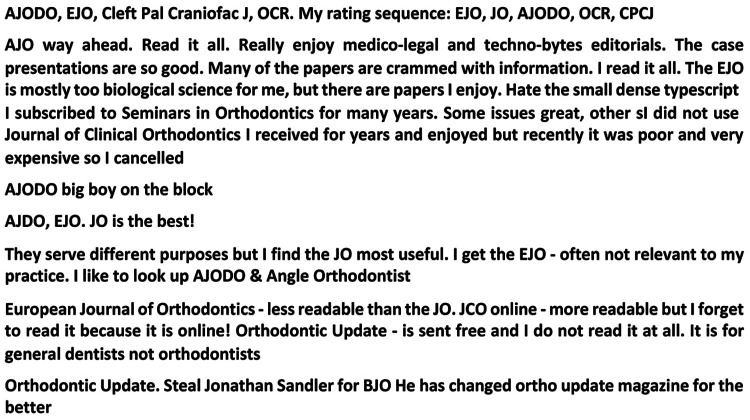
Do you receive any other orthodontic journals? A sample of representative comments (from a total sample of 175 responses).

The survey went on to ask if responders received any other orthodontic journals with the majority of 68.7% (n = 213) answering that, yes, they did and 31.3% (n = 97) saying that they did not. If they did receive other orthodontic journals, they were further asked to state what they were and a total of 175 individuals did this (Table 2). A number of these individuals made further comments in relation to this question and a selection of these is included in Figure 6.

**Table 2.** Other journals received by respondents.

*Orthodontic Update*	85
*American Journal of Orthodontics and Dentofacial Orthopedics*	40
*European Journal of Orthodontics*	33
*Journal of Clinical Orthodontics*	15
*British Dental Journal*	5
*Angle Orthodontist*	4
*World Federation of Orthodontists*	3
*Australian Orthodontic Journal*	3
*Orthodontics and Craniofacial Research*	2
*Seminars in Orthodontics*	1
*Cranio UK*	1
*Cleft Palate Craniofacial Journal*	1

**Figure 7.**
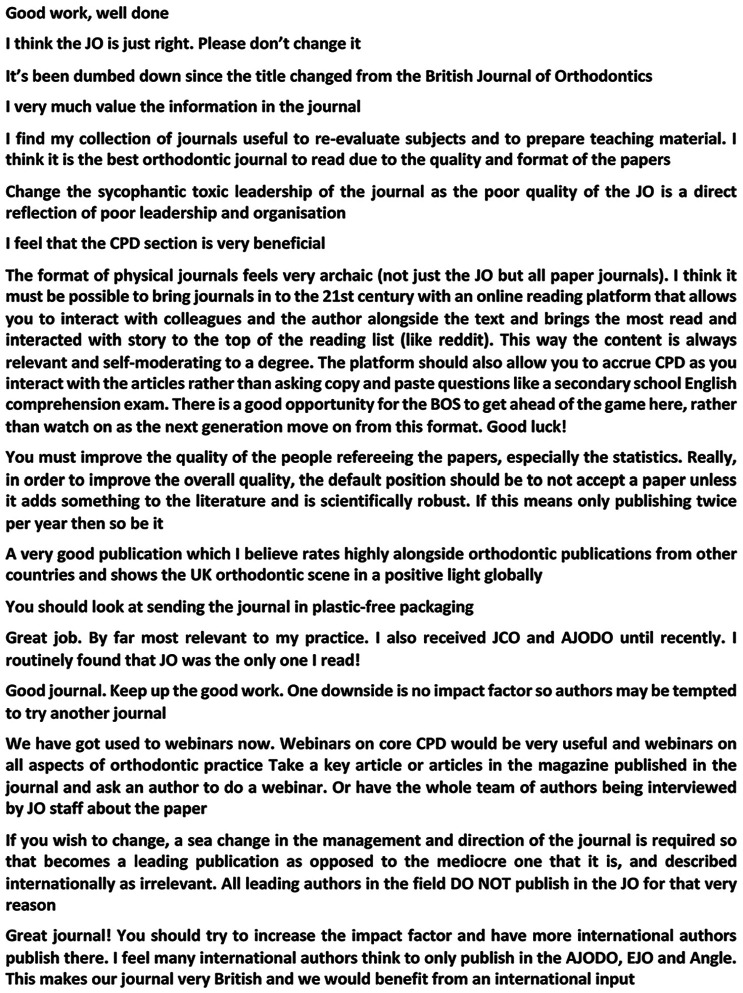
A representative sample of general comments (from a total sample of 310).

The final question related to general feedback and all 310 respondents made at least one comment. A selection of these comments is reproduced in Figure 7.

## Discussion

Reading an academic journal gives the clinician an opportunity to engage in current thinking and develop skills within their field of expertise (Tenopir et al., 2007). We have carried out a survey of members of the British Orthodontic Society and canvassed their opinion on the *Journal of Orthodontics*, which is the representative academic journal of their society. This is the first time that the readership of the *Journal* has been asked directly to give their opinion on content and potential future direction relating to this publication. In broad terms, the level of satisfaction with the *Journal* was good among the readership, which suggests that the *Journal* is fulfilling its essential remit for the British Orthodontic Society. This is consistent with previous readership surveys of both dental and medical journals (Brochard, 2005; Carroll, 2017; Stott, 2003). The vast majority of respondents rated the *Journal* positively and felt that it was relevant to their clinical practice. Moreover, most people read at least one article per issue and the scientific and clinical articles were generally reported as being the most popular.

In general, respondents liked to read the *Journal* in a traditional printed format although some did choose to access the digital version. The preference of a print rather than digital journal is not unusual (Ronellenfitsch et al., 2015; Tenopir et al., 2007). The perceived benefits of a printed format include superior quality, allowing the inclusion of higher quality images, convenience of access and reading, and the facility for the reader to make notes directly while reading (Tenopir et al., 2007). In contrast, the benefits of digital journals have been suggested to be better usage, ease of access, printing and searching (Rutkowski and Dohan Ehrenfest, 2012; Sathe et al., 2002). Although not explored in this survey, previous investigators have reported that the preference of journal format may be an age-related phenomenon (Ronellenfitsch et al., 2015) and influenced by different age groups’ usage of electronic devices (Tenopir et al., 2007). Interestingly, opinion was divided on the need for an increased number of issues per year or, indeed, the production of additional supplements. The *Journal* routinely carries advertisements that are placed by the British Orthodontic Society and these were generally viewed in a positive light.

The survey questions were put together by the authors in consultation with the editorial board and while most of the questions were binary in nature, opportunity was given for respondents to provide further thought on some of the themes within free-text boxes. There were over 500 of these individual responses and much further information was obtained from them. The veil of anonymity provided by the survey did allow some individuals to be quite robust in their rhetoric and it can be seen that some opinions were quite forceful, particularly from those who are less happy with the *Journal* and the society it represents. Moreover, in some circumstances individual comments related to the same question were polar opposites of each other, which largely demonstrates that, of course, it is impossible to please everybody all of the time. However, analysis of these comments did allow some themes to emerge and they provide some useful things to consider adopting for the *Journal* as it moves forward.

One theme was the feeling that the *Journal* is too academic, that often the research articles engage subjects lacking relevance for many orthodontists and that there should be more clinical content, demonstrating new ideas and techniques that are applicable to routine orthodontics. Academic medical journals have been criticised for lacking articles that are relevant to practising clinicians (Benson, 2008). Indeed, a desire for practical clinical techniques and ‘How to do it’ articles from the readership of specialty journals has been previously reported (Stott, 2003). This does result in something of a paradox. In an age of evidence-based medicine, publishing high-quality clinical research studies are encouraged but clinicians seem to prefer lower-level case reports and descriptive clinical articles. It could also be argued that a desire for more clinically relevant articles might be related to the highest number of respondents being specialist practitioners. Moreover, the timing of the survey coincided with a return to clinical activity following the lifting of COVID-19 restrictions in the dental setting and this may also have influenced a desire for clinical content. In the assessment of articles published in four orthodontic journals (*American Journal of Orthodontics and Dentofacial Orthopedics, Angle Orthodontist, European Journal of Orthodontics* and *Journal of Orthodontics*) between 1999 and 2008, around half the articles published in the *Journal of Orthodontics* reported treatment of human subjects with at least 20% of each edition containing case reports (Gibson and Harrison, 2011). In addition, more materials-based and animal experiment articles tended to be published in the *Angle Orthodontist* and *European Journal of Orthodontics*, respectively (Gibson and Harrison, 2011). Based on these findings, the perception that the *Journal of Orthodontics* is ‘too academic’ may be unfounded. Despite this, it appears that not all clinical articles and their content appeal to everyone. This is highlighted by free-text comments such as ‘*More practice-based clinical pearls*’ and ‘*Slightly more clinically relevant articles focusing on all treatment modalities – aligners/ceramics, etc., to broaden our horizons – not just NHS-funded treatments*’. To address this type of need, some journals have clinical case letter sections, which aim to inform readers of new or alternative clinical techniques or interesting clinical situations (Rutkowski and Dohan Ehrenfest, 2012).

Another suggestion was the incorporation of more reader interaction with the *Journal*, perhaps through webinars with authors and by expanding the continued professional development associated with published articles. There was a feeling from some that many papers published by the *Journal* are often from the same authors and that subject areas can be repetitive. When considering how the *Journal* is placed in relation to other international orthodontic journals there was divided opinion, with some respondents suggesting that it needs to develop a more international focus rather than being a ‘society’ journal. Among other orthodontic journals that are published, the *American Journal of Orthodontics and Dentofacial Orthopedics* was held in high overall regard, while the *European Journal of Orthodontics* was also popular, albeit with the caveat that it tends to publish a lot of biological articles. Interestingly, *Orthodontic Update* received a lot of positive comments, the clinical-focussed content being popular with readers. A number of respondents raised the issue of the *Journal of Orthodontics* lacking an impact factor, which they perceived as being a barrier to its further development. They suggested that many United Kingdom academic orthodontists publish their work in other orthodontic journals for this reason. There may be some truth in this statement, and certainly the lack of impact can be a barrier to receiving the best submissions, although it is questionable whether many universities would use articles published in any of the orthodontic specialty journals for a research assessment exercise. It should also be remembered that a journal impact factor is not the only metric of academic rigour and over the years, through the efforts of previous editors, such as Kevin O’Brien and Philip Benson, the *Journal of Orthodontics* has been proactive in encouraging high reporting standards for its articles (Koletsi et al., 2012). Moreover, journal impact factors are susceptible to manipulation and multiple strategies are available for journals to game the system (Ioannidis and Thombs, 2019). Impact factors rely on citation counts, journals can encourage citation of their own publications, they can publish multiple review articles that are generally more likely to be cited and they can publish articles open access to increase their reach and the likelihood of being cited. There are no such editorial or publishing strategies in place at the *Journal of Orthodontics*.

### Limitations of the survey

It was decided to maximise anonymity in association with the survey to encourage honest and wide-ranging responses, which meant only limited demographic data were available. There are sources of bias associated with this survey, not least the relatively low response rate from the membership. Given the previous trend of low response rates to British Orthodontic Society member-surveys on subjects such as orthodontic treatment planning and training experiences (Fleming et al., 2018; Oliver et al., 2020), strategies should have been introduced to try and improve this. The developers of future surveys of this membership group should take this into consideration at the planning stage. It is the opinions of the more hard-to-reach groups that are potentially the most interesting, otherwise a respondent bias is introduced. It might have been prudent to pilot the questionnaire first, rather than developing it only in consultation with the editorial board to increase potential relevance for the membership. Indeed, it could be argued that this survey should be used as a basis for future investigators to refine the question-base and attempt the deployment of strategies to reach more non-responders. In addition, the introduction of methods other than questionnaires should also be considered, to allow a more inquisitive approach to data acquisition.

## Conclusion

This survey has provided some insight into the opinion of members of the British Orthodontic Society who receive the *Journal of Orthodontics*. The *Journal* is perceived as being relevant to current clinical practice and has high levels of satisfaction. However, there was some desire for more online interaction between the membership and *Journal* as part of its role within the society. These conclusions should also be considered within the context of a low response rate and the existence of potential bias.

## Supplemental Material

sj-docx-1-joo-10.1177_1465312520988549 – Supplemental material for The *Journal of Orthodontics*: A cross-sectional survey of British Orthodontic Society members Click here for additional data file. Supplemental material, sj-docx-1-joo-10.1177_1465312520988549 for The *Journal of Orthodontics*: A cross-sectional survey of British Orthodontic Society members by Rosalind Jennings, Jadbinder Seehra and Martyn T Cobourne in Journal of Orthodontics
