# Supplementary material for: The Journal of Orthodontics: A cross-sectional survey of British Orthodontic Society members
Source: J Orthod. 2021 Feb 5;48(2):101–9. doi: 10.1177/1465312520988549 (PMC8225694; doi:10.1177/1465312520988549)
Supplement: sj-docx-1-joo-10.1177_1465312520988549 – Supplemental material for The Journal of Orthodontics: A cross-sectional survey of British Orthodontic Society members [file sj-docx-1-joo-10.1177_1465312520988549.docx]

**Appendix *Journal of Orthodontics* Reader questionnaire**

1. **What is your role?**

- Specialist practitioner
- Consultant orthodontist
- Orthodontic registrar
- Orthodontic therapist
- Orthodontic nurse
- Orthodontic technician
- Other (please specify)

1. **How often do you read the *Journal of Orthodontics*?**

- I usually read at least one article in every edition
- I read articles in a couple of editions per year
- I read articles in one edition per year
- I open it and just look at the table of contents
- It arrives and I don’t even open it

1. **Would you like to see a copy published every two months?**

- Yes
- No

1. **How do you read the *Journal of Orthodontics*?**

- Hard copy
- Digital copy
- Both
- Neither

1. **Would you prefer only to receive a digital copy?**

- Yes
- No

1. **How would you rate the *Journal of Orthodontics*?**

- Excellent
- Satisfactory
- Poor
- Please state the reason for this rating.

1. **What do you like best about the *Journal of Orthodontics* content? Tick all that apply.**

- Scientific papers
- Clinical papers
- CPD
- Abstract reviews
- Statistical corner
- None of them interest me very much
- Other (please specify)

1. **What do you dislike about the *Journal of Orthodontics*? Tick all that apply.**

- Scientific papers
- Clinical papers
- CPD
- Abstract reviews
- Statistical corner
- None of the above
- Other (please specify)

1. **Would you like to see more supplements?**

- Yes
- No
- Comments

1. **Do you have a favourite supplement from the last few years?**

- Yes
- No
- If yes, please specify.

1. **Do you feel that the *Journal of Orthodontics* is relevant to your clinical practice?**

- Yes
- No
- Please state the reason for your answer.

1. **How do you feel about the advertisements in the Journal?**

- I like the advertisements, they are useful
- I do not like the advertisements
- I do not think the Journal should have commercial advertisements

1. **Is there anything specific that you would like to see changed in the *Journal of Orthodontics*? Tick all that apply.**

- I would like to see a regular editorial
- I would like to see editorials by guest authors
- Scientific papers
- Clinical papers
- Features
- No changes are necessary
- Other
- Comment

1. **Do you receive any other orthodontic journals?**

- Yes
- No
- If you receive other orthodontic journals, please state what they are and how they rate in comparison to the Journal of Orthodontics.

1. **We are very interested in any other feedback relating to the *Journal of Orthodontics*. Please write any other comments or feedback that you may have below.**
